# Age and Sex Influence the Neutrophil-to-Lymphocyte Ratio (NLR), a Systemic Inflammation Marker

**DOI:** 10.3390/clinpract16100178

**Published:** 2026-09-24

**Authors:** Carmen Lara-Apolinario, Cesar Martinez-Carral, Desiree Fischetti, Adexe J. Fulgencio-González, Casimira Domínguez, Pedro C. Lara

**Affiliations:** 1 Preventive Medicine and Public Health Department, Infanta Elena University Hospital, 28342 Madrid, Spain; cala.edu@quironsalud.es; 2 Medicine Department, Fernando Pessoa Canarias University, 35450 Guía, Spain; 3 Preventive Medicine and Public Health Department, Cruces University Hospital, 48903 Barakaldo, Spain; 4 Laboratory Medicine Department, Dr Negrín University Hospital, 35010 Las Palmas de Gran Canaria, Spain; 5 Canarian Comprehensive Cancer Center, San Roque University Hospital, 35001 Las Palmas de Gran Canaria, Spain; 6 Canarian Institute of Cancer Research, 38320 La Laguna, Spain

**Keywords:** NLR, controls, age, sex, European

## Abstract

**Background/Objectives**: The neutrophil-to-lymphocyte ratio (NLR), as a marker of systemic inflammation, has been studied in a wide variety of diseases. Unfortunately, there are very limited data describing the NLR distribution and reference intervals for a normal population in specific geographical areas. The aim of this study was to analyze, for the first time, different patterns in NLR distribution, stratified by age and sex, in a large group of controls from a European country. **Methods**: Patients referred for a health examination to the Laboratory Department of Dr Negrín University Hospital from 1 January 2021 to 31 December 2025 were included in this study. The participants had normal hematological values, lipid metabolism markers and hepatic enzymes, erythrocyte sedimentation rates (ESRs), and C-reactive protein (CRP) levels. Samples were treated anonymously, including only sex and date of birth. The neutrophil-to-lymphocyte ratio (NLR) was determined by dividing the absolute neutrophil count by the absolute lymphocyte count. **Results**: A total of 30,676 controls were included in the study. NLR increased with age (*p* < 0.0001). Women showed lower NLR values than men (*p* < 0.0001), revealing an age-related pattern, with values increasing until age 45, dropping from 46 to 65, and slightly increasing in advanced age (>65 years). **Conclusions**: To our knowledge, this is the largest study from a European country analyzing the impact of sex and age on NLR values in controls. An interesting differential pattern of NLR modifications according to age group was observed in women. Therefore, NLR values must always be interpreted according to sex and age groups.

## 1. Introduction

The neutrophil-to-lymphocyte ratio (NLR), as a marker of systemic inflammation, has been widely studied in cardiovascular diseases [1,2,3,4], infection [5], autoimmune diseases [6,7,8,9,10,11], cancer [12,13,14,15,16,17,18,19,20,21,22,23,24,25,26], adiposity-based chronic disease [27], diabetes [28], liver disease [29], depression [30,31], addiction [32], neurological disorders [33,34], and dementia [35].

There are limited data from large population series describing the NLR distribution for a normal population [36]. The seminal study of Azab et al. [37] analyzed 9427 subjects included in the National Health and Nutrition Examination Survey (NHANES) from 2007 to 2010. The average NLR was 2.15. Non-Hispanic Black and Hispanic participants had significantly lower mean NLR values when compared to non-Hispanic White participants.

Forget et al. [38] described NLR values in a series of 413 healthy young adult controls in France. The mean NLR was 1.65 [±1.96 SD: 0.78–3.53] (95% CI [0.75–0.81]. They proposed normal NLR values of between 0.78 and 3.53 for an adult, non-geriatric population in good health.

The largest study in Hispanic healthy controls was performed by Huguet et al. in Argentina [39]. They included a total of 71,873 subjects ranging from 16 to 103 years of age. The median NLR varied from 1.52 to 2.44. The NLR increased with age (1.52 in the 20s to 2.44 in the 90s), and women had lower NLR values than men.

Another large South Korean study [40] included 12,160 samples from healthy controls. The mean NLR across all ages was 1.65 (0.79), and the values for men and women were 1.63 ± 0.76 and 1.66 ± 0.82, respectively. The results suggested that different cutoff values should be applied to different patient populations.

Several studies have been conducted in different regions of China [41,42,43]. The earliest study by Meng et al. [41] included 24,029 samples from healthy donors in central China. They found that the NLR was significantly different between gender (*p* = 0.010) and age (*p* < 0.001) groups. A second study in western China including 5969 healthy controls from the Han ethnic group [42] found that neither gender nor age influenced the NLR (*p* > 0.05).

The most recent study from China [43] included a total of 165,504 healthy controls. Female participants had a mean NLR of 0.04, higher than the male participants (*p* < 0.0001). Age was associated with an increase in NLR.

The only available study in Spain used the Aging in Spain Longitudinal Study (ELES Study), a cross-sectional study designed to collect health data to analyze the aging process. The study included only noninstitutionalized persons aged 50 years or older. A total of 1747 older adults were included (771 men and 976 women). The mean age of the sample was 66.2 ± 10.64 years. The mean NLR for the whole group was 2.03 ± 0.89: 2.15 ± 0.96 for men and 1.93 ± 0.82 for women (*p* < 0.001) [44].

From a biological point of view, aging causes a chronic, low-grade inflammatory phenotype termed “inflammaging” [45], primarily through marked alterations in adaptive immunity, including a decline in circulating lymphocytes that is largely attributed to reduced thymic activity [46]. In contrast, peripheral neutrophil production and circulation remain stable or slightly elevated, increasing the ratio’s numerator while the lymphocyte denominator falls [45].

It is well-known that there are sex differences in the immune response, especially the adaptive immune response [47]. These differences may be explained by age-related changes in hormonal levels and the induced re-shaping of the immune system [48].

Controversial results observed in series regarding the influence of age and sex on NLR values have been described above. Most [39,42,43] but not all [38,40,42,44] of those series demonstrated an increase in NLR with increased age across the whole patient series or in some subgroups (non-Hispanic White participants) [37]. Furthermore, men showed higher NLR values than women in some studies [41,44], while no relation was found in others [39,44]; some studies even found a higher NLR in women compared to men [39,40,43].

As described above, studies from European countries [38,44] did not analyze age in relation to NLR values, and only one [44] analyzed sex variations.

There is strong evidence suggesting that NLR has a prognostic role in a wide variety of medical conditions. There is a need to analyze NLR values according to age and sex within a homogeneous population. The limited evidence available from European cohorts [38,44] highlights the need for a large study including participants from a European country.

The aim of the present study was to analyze, for the first time, patterns of NLR distribution stratified by age and sex in a large group of controls from a European country.

## 2. Methods

Patients referred to the laboratory Department of Dr Negrín University Hospital, from 1 January 2021 to 31 December 2025 for a health examination were included in this study. Study inclusion criteria were normal hematological values (neutrophils: 1500–10,000/mm^3^; lymphocytes: 1500–4500/mm^3^), lipid metabolism markers (cholesterol: 0–200 mgr/dL; triglycerides: 35–150 mgr/dL), hepatic enzymes (ALT: 0–40 μ/L; ALP: 0–45 μ/L), erythrocyte sedimentation rate (ESR, 0–20 mm/h), and C-reactive protein (CRP, 0.00–5.00 mgr/L). Candidate cases with concomitant positivity for cocaine, amphetamine/methamphetamine, cannabis, or opioids were excluded due to the impact of these substances in NLR values [34]. Control participants were aged 18 years and above. Samples were treated anonymously, including only sex and date of birth; therefore, no information on possible comorbidities was available.

Blood samples were collected after overnight fasting for 12 h. Based on electrical impedance, flow cytometry, and optical light scattering, a CELL-DYN Sapphire hematology analyzer (Abbott Diagnostics, Abbott Park, IL, USA) was used to determine complete blood cell counts and differential counts of white blood cells in anticoagulated whole blood. The neutrophil-to-lymphocyte ratio (NLR) was determined by dividing the absolute neutrophil count by the absolute lymphocyte count.

Drugs of abuse were detected in urine using an automated Alinity C analyzer (Abbott Laboratories, Abbott Park, IL, USA) and homogeneous immunoassays with the manufacturer’s commercial reagents. The toxicological panel included amphetamines/methamphetamines, cocaine (benzoylecgonine metabolite), cannabinoids (THC-COOH) and opiates. Results were reported qualitatively (positive/negative), using cut-off values of 50 ng/mL for cannabinoids, 300 ng/mL for cocaine and metabolites, 500 ng/mL for amphetamines and derivatives, and 300 ng/mL for opiates.

### 2.1. Ethics

This study was conducted in accordance with the principles of the Declaration of Helsinki and approved by the Ethics Committee of the Fernando Pessoa University on 24 June 2024 (EC code: PI 005-2024) and the Ethics Committee of the Doctor Negrín University Hospital on 26 February 2026 (EC code: 2026-082-1). Considering that the analysis was retrospective and that the samples were collected, analyzed, and treated anonymously, the ethics committees waived the need for individual consent. Consequently, no written informed consent to participate in the study was obtained from the participants.

### 2.2. Statistical Analysis

Study variables’ distributions were tested for normality (Kolmogorov–Smirnov test). Bivariate analysis correlations were performed according to Pearson’s test for normally distributed variables and Spearman’s test for non-normally distributed variables. Continuous variables compared to gender or age intervals were analyzed using the appropriate test (*t*-test for normally distributed variables and the Wilcoxon/Kruskal–Wallis test for non-normally distributed variables). A probability of 0.05 was considered statistically significant. Statistical analyses were performed using SPSS v26 (IBM Corp., Armonk, NY, USA).

Categorical variables are expressed as frequencies and proportions, while continuous variables are presented as means and medians with interquartile range (IQRs). Interquartile range values of Q25% and Q75% are shown. The pre-specified age groups were 18–25, 26–35, 36–45, 46–55, 56–65, and >65 years. For data not following a normal distribution, non-parametric tests were used for analysis. Non-parametric rank correlation analysis was employed for linear correlation analyses. A probability level of 0.05 was considered statistically significant.

## 3. Results

From 1 January 2021 to 31 December 2025, a total of 30,381 controls met the selection criteria out of 1,172,990 blood test results screened. There was 16,555 women (54.49%) and 13,826 men (45.51%) (*p* < 0.0001). More than 70% of the controls were older than 36 y (Table 1). The mean age of participants was 54.03 ± 17.24 y (range: 18–103).

### 3.1. Neutrophil and Lymphocyte Counts

Neutrophil counts were available for all participants: the mean value was 4.10 ± 1.25 × 10^3^/mm^3^ (range 1.57–8.93), the median was 3.90 × 10^3^/mm^3^, and the IQR was 3.16–4.84 × 10^3^/mm^3^. Lymphocyte counts were also available for all patients: the mean value was 2.26 ± 0.54 × 10^3^/mm^3^ (1.50–4.50), the median value was 2.15 × 10^3^/mm^3^, and the IQR was 1.84–2.56 × 10^3^/mm^3^. Both neutrophil and lymphocyte counts followed a non-normal distribution. The neutrophil counts increased with age (*p* < 0.0001), while the lymphocyte counts decreased with age (*p* < 0.0001) (Figure 1).

### 3.2. Neutrophil-to-Lymphocyte Ratio (NLR) and Age

The NLR was determined by dividing the absolute neutrophil count by the absolute lymphocyte count. The NLR mean was 1.88 ± 0.64 (range: 0.94–4.96), and the median was 1.73 (IQR: 1.39–2.20). The NLR values in our series of participants followed a non-normal distribution.

There was a significant increase (Spearman’s Rho: 0.111; *p* < 0.0001) in the NLR with increased age in the bivariate analysis (Figure 2). When age was stratified in prespecified age groups, a significant increase in NLR through the different age groups was observed. The NLR ranged from a mean of 1.70 and median of 1.56 (IQR: 1.30–1.95) in controls aged 18 to 25 to a mean of 1.98 and median of 1.82 (IQR: 1.44–2.36) in those >65 y (*p* < 0.0001) (Figure 3).

### 3.3. Neutrophil-to-Lymphocyte Ratio (NLR) and Sex

Men had higher NLR values (mean: 1.93 ± 0.67; median 1.77; IQR: 1.42–2.29) than women (mean: 1.83 ± 0.62; median: 1.69; IQR 1.37–2.14; *p* < 0.0001) (Table 2). The NLR showed higher increases with age in men (Spearman’s Rho: 0.197) compared to women (Spearman’s Rho: 0.032; Fisher’s Z-transformation test, Z: 14.39; *p* < 0.0001) (Figure 2B,C).

When age was stratified in the prespecified age groups, distributions differed for men and women (Figure 3B,C). In fact, men showed a continuous increase in NLR through the age groups (Table 2, Figure 3B), while women seemed to show an age-related pattern (Table 2, Figure 3C). Compared to men, there was a faster increase in the NLR in women at younger ages, with a peak in the 36–45 y decade and overall higher NLR values than men up to that age range (Table 2). This was followed by a decrease in the NLR in the 46–55 y and 56–65 y groups and another increase at older ages (>65 y). After 46 y, the NLR was statistically higher in men than in women (Table 2).

To explain the factors inducing these different patterns, we performed a detailed exploratory analysis of the neutrophil and lymphocyte counts stratified by age group.

The neutrophil count correlated directly with advanced age (Spearman’s Rho: 0.065; *p* < 0.0001). Conversely, the lymphocyte count was inversely related to age (Spearman’s Rho: −0.077; *p* < 0.0001). Neutrophil counts increased with age in men (Spearman’s Rho: 0.147; *p* < 0.0001) but not in women (Spearman’s Rho: −0.011; *p* = 0.151). Lymphocyte counts decreased with age in both men (Spearman’s Rho: −0.097; *p* < 0.0001) and women (Spearman’s Rho −0.063; *p* < 0.0001). Neutrophil counts in men showed a mean of 4.20 ± 1.25 (median 4.02, IQR: 3.26–4.97) × 10^3^/mm^3^. higher than the mean of 4.01 ± 1.23 (median: 3.81, IQR: 3.10–4.73) × 10^3^/mm^3^ observed in women (*p* < 0.0001). Lymphocyte counts were similar in men (mean: 2.26 ± 0.54, median: 2.15, IQR: 1.84–2.57) × 10^3^/mm^3^ and in women (mean: 2.25 ± 0.54, median: 2.15, IQR: 1.84–2.56) × 10^3^/mm^3^ (*p* = 0.721).

Figure 4 depicts the whole series of participants, stratified according to pre-defined age and sex groups. Men showed a continuous increase in neutrophils and decrease in lymphocytes with age (Figure 4A–C). On the contrary, women showed a varying pattern in neutrophil counts across the age ranges (Figure 4B). Like in men, lymphocyte counts in women decrease with age (Figure 4D).

## 4. Discussion

We present a detailed analysis of the influence of age and sex on the NLR in the largest series of patients studied in Europe. Increasing age was related to increased NLR in the whole-population analysis, but very different patterns of NLR distribution were observed among men and women with increasing age.

A detailed analysis of the published evidence regarding reference intervals for NLR in healthy control populations (Table 3) showed that most [39,41,43], but not all [38,40,42,44], of those series demonstrated an increase in the NLR with increased age in whole patient series or in some subgroups (non-Hispanic White controls) [37]. It is noteworthy that in the two available European studies, the possible influence of age on the NLR was not analyzed [38,44].

Our results showed, in a large series of controls from a European country, that the NLR may increase with age. Considering our results and those of others [48], the changes observed in the NLR with increased age may be related to immunosenescence [45].

Aging is accompanied by marked alterations in adaptive immunity, including a decline in circulating lymphocytes that is largely attributed to reduced thymic activity [49]. Peripheral T lymphocytes comprise a diverse population that includes naïve, effector, and memory subsets. Highly differentiated T cells progressively acquire characteristics associated with cellular senescence, including diminished proliferative capacity, telomere shortening, and increased activation of the p38 signaling pathway [50]. B lymphocytes likewise develop senescence-related characteristics, primarily reflected by the acquisition of a pro-inflammatory senescence-associated secretory phenotype [51]. Furthermore, a distinct population of age-associated B cells (ABCs), which expands during aging, has emerged as an important contributor to immunosenescence. These cells are present at increased frequencies in autoimmune and autoinflammatory disorders [52]. Neutrophils constitute a major component of the innate immune system and serve as the primary defense against bacterial pathogens and other diseases such as cancer [53]. Unlike lymphocytes, neutrophil counts remain relatively stable during immunosenescence [54]. Senescent neutrophils exhibit several hallmark features of cellular aging, including telomere attrition, diminished proliferative potential, and the development of a pro-inflammatory senescence-associated secretory phenotype [55].

In our study, circulating lymphocyte counts progressively declined with advancing age, whereas neutrophil numbers increased over time (Figure 1). Therefore, the neutrophil-to-lymphocyte (NLR) ratio rose with age (Figure 3). 

Sex differences in the NLR were observed in our study. Men showed higher NLR values compared to women. This fact has also been observed in other published series [41,44], although some other studies did not show a significant difference in the NLR according to sex [37,42]; some even observed a higher NLR in women compared to men [39,40,46]. Regardless, the reported differences in the NLR between men and women were small, ranging from 0.01 [41] to 0.08 [37]. Data from the Spanish [44] study clearly fell outside the published range (0.22), although the reported data reflected mean and not median values. In our study, the median difference observed was 0.10, well within the range reported in other published studies.

In an attempt to understand these controversial results, we observed a different patterns of the NLR increasing with age between men and women. Men demonstrated higher increases in the NLR with age (Spearman’s Rho: 0.197) compared to women (Spearmen’s Rho: 0.032). When age was stratified into prespecified age groups, men and women showed different patterns of NLR distribution. Specifically, men showed a continuous increase in the NLR through the different age groups, with an almost linear increase in neutrophils and a parallel decrease in lymphocyte counts (Figure 4).

Women show a different age-related pattern. They exhibited a faster increase in the NLR compared to men, peaking in the 36–45 y decade. This was followed by a decrease in the NLR for the 46–55 y and the 56–65 y ranges and another increase in the NLR at older ages (>65 y). This pattern was primarily based on the neutrophil increase in the younger age groups followed by the decrease in middle age and a further increase after 65 years. The observed decrease in lymphocytes also differed from that seen in men, with a lower linear correlation in women.

Similar results were obtained by Huguet et al. [36] in a Latin American population, Lee et al. [40] in South-Korea, and Wang et al. [39] in China. In this study, we present for the first time similar data in a population from a European country. This distinctive changes in the NLR with age in women could explain the controversial results obtained in other studies regarding the influence of sex on NLR values. Our results and others clearly demonstrate that NLR values in women are highly dependent on age [36,39,40].

Sex differences in the immune response are well-known [56], as women seem to have better innate and adaptive immune responses than males, counterbalanced by their increased susceptibility to inflammatory and autoimmune diseases [57,58].

Absolute lymphocyte counts did not differ between sexes in our study (*p* = 0.721), but male vs. female dimorphism was observed for the neutrophil count (*p* < 0.0001) [59]. These differences could be explained by age-related changes in hormonal levels and the induced re-shaping of the immune system [47]. In a recent study, premature ovarian insufficiency (POI) was associated with a sudden drop in NLR values [60], highlighting the role of hormones, especially estradiol, in the increasing levels of neutrophils detected [61].

Our results shed light on NLR values in the largest series to date of participants from a European country. When NLR values for controls are proposed, they should take into account sex and age groups; therefore, any study examining the NLR in a certain disease must include a clear description of the age and sex of the cohort studied for comparison with published data on healthy controls of the same age and sex. If possible, there should also be a large, well-paired group of healthy controls to ensure comparability between cases and controls.

### Limitations

Our study has several limitations. While normal hematological, lipidic, liver and toxicology data were available for our controls, no data on comorbidities, medication use, or lifestyle factors were accessible due to Spanish legislation protecting personal data in the possession of private parties. Additionally, the number of participants is lower than in other series from China and Latin America.

## 5. Conclusions

To the best of our knowledge, this is the largest European study analyzing the impact of sex and age on NLR values in controls. An interesting differential pattern in NLR changes according to age was observed in women. As NLR values may be modified by age and sex characteristics, studies analyzing the role of NLR in the context of specific medical conditions should include a large, well-paired group of healthy controls to ensure the comparability of the NLR values between cases and controls.

## Figures and Tables

**Figure 1:**
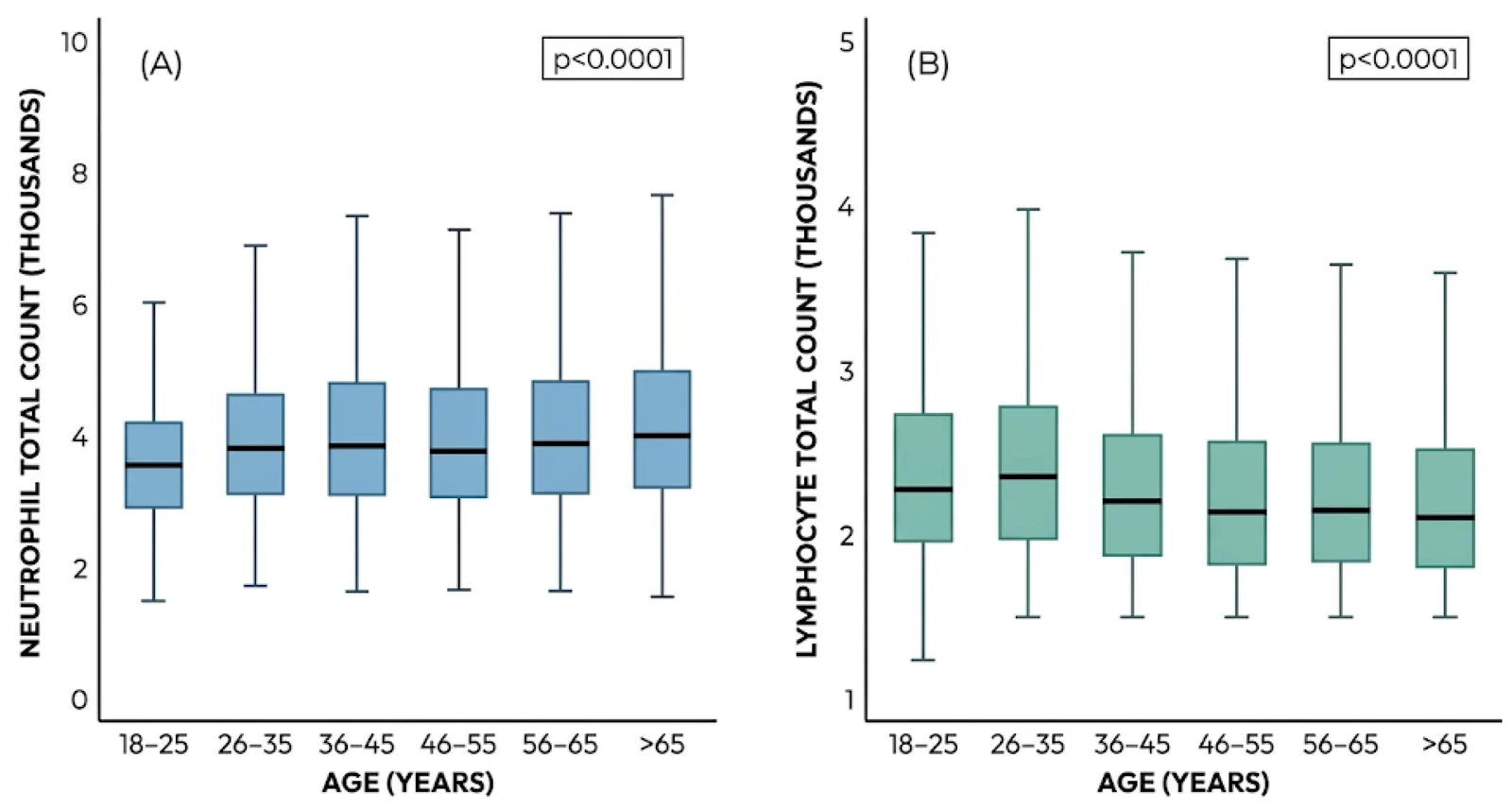
Neutrophil and lymphocyte counts related to age. (**A**) Neutrophils; (**B**) Lymphocytes.

**Figure 2:**
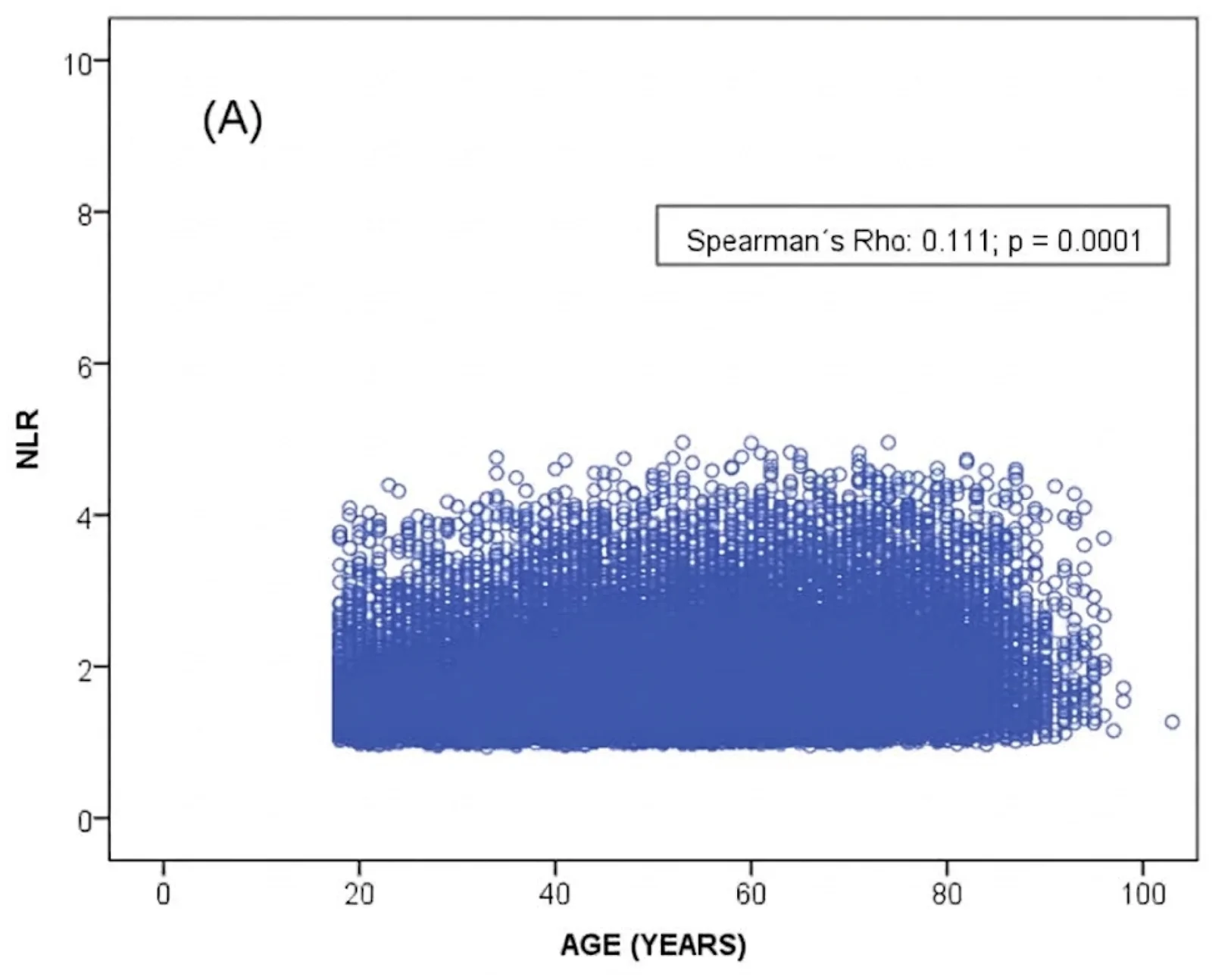

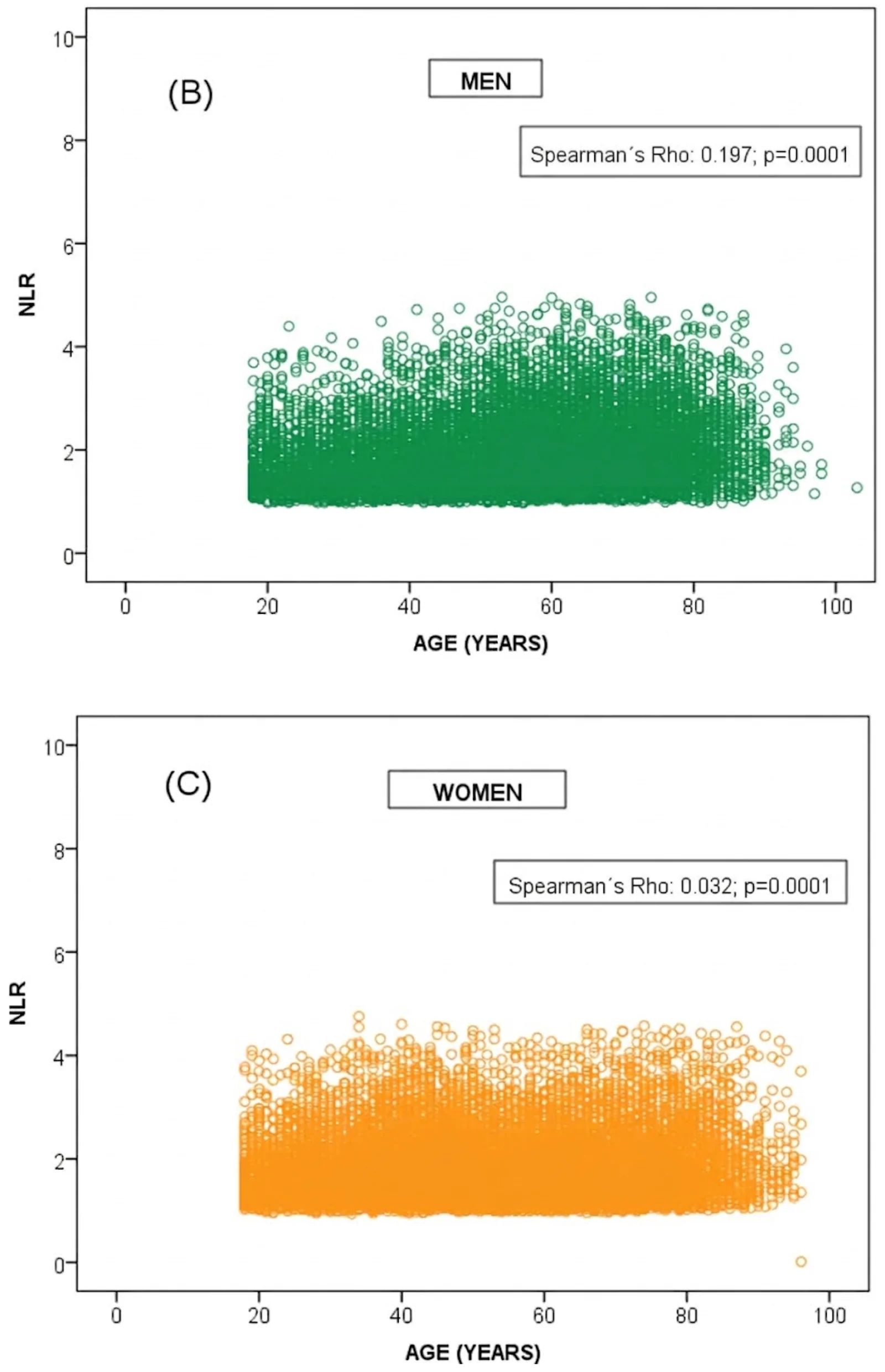
Age and NLR correlation: (**A**) whole series; (**B**) men; (**C**) women.

**Figure 3:**
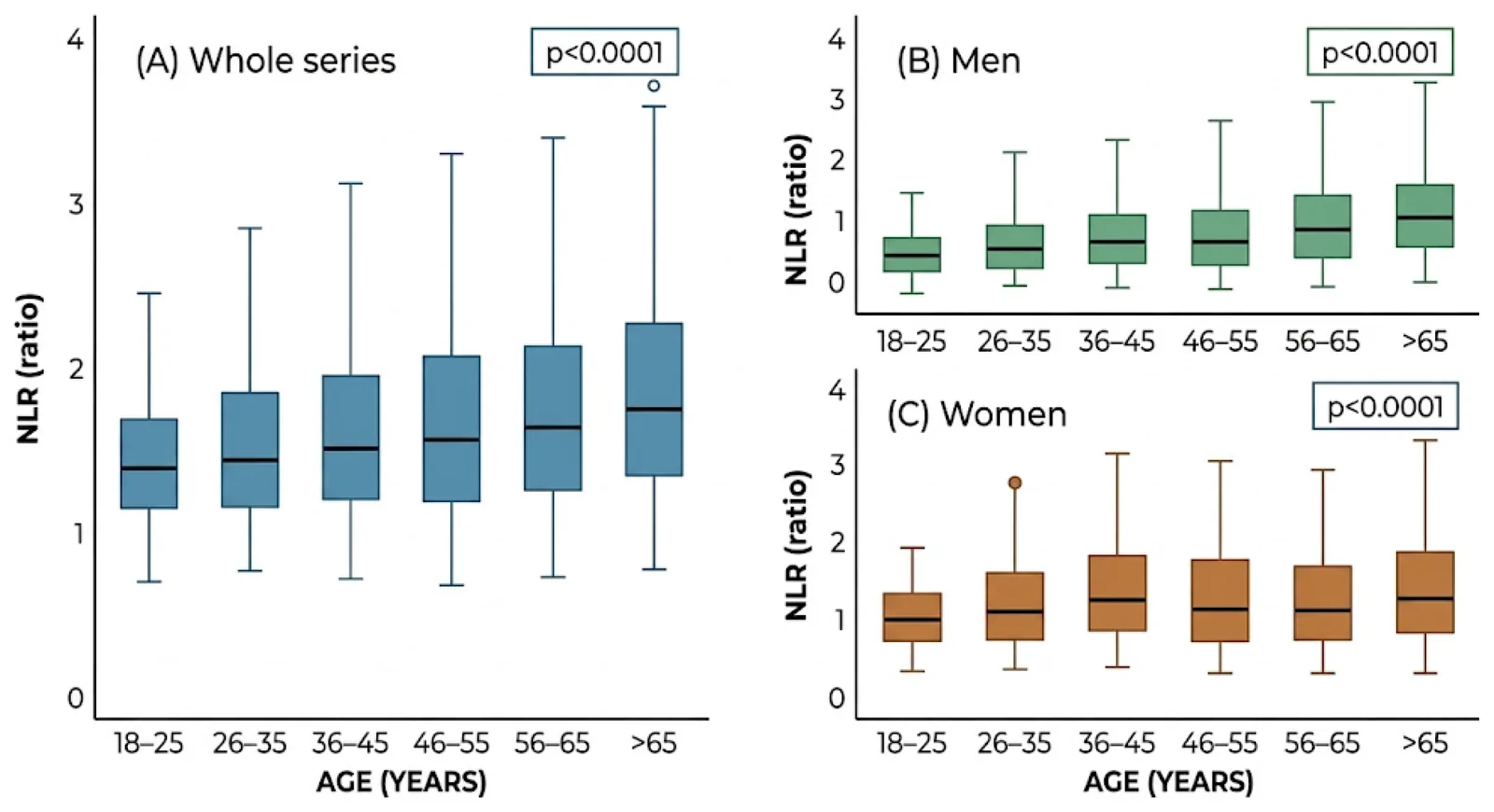
NLR distribution according to age groups: (**A**) whole series; (**B**) men; (**C**) women.

**Figure 4:**
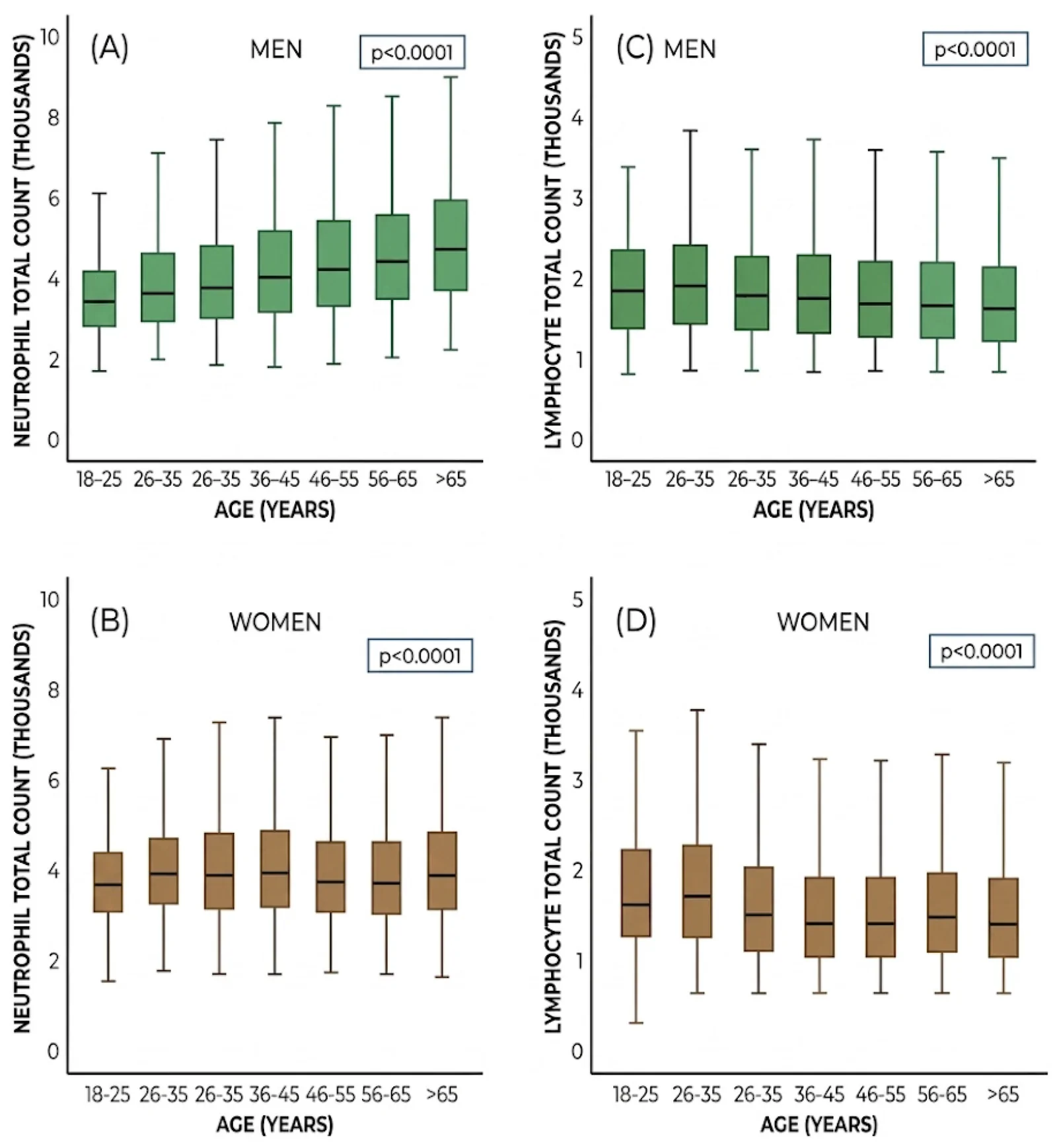
Neutrophil (**A**) men, (**B**) women and lymphocyte counts (**C**) men, (**D**) women, according to sex and age groups.

**Table 1:** Control distribution by age and gender.

Age (Years)	Women	Men	Total
18–25	1061 (56.77%)	808 (43.23%)	1869 (6.15%)
26–35	1926 (59.06%)	1335 (40.94%)	3261 (10.73%)
36–45	2943 (63.31%)	1706 (36.69%)	4649 (15.30%)
46–55	3071 (54.22%)	2593 (45.78%)	5664 (18.64%)
56–65	3040 (47.90%)	3307 (52.10%)	6347 (20.89%)
>65	4514 (52.54%)	4077 (47.46%)	8591 (28.27%)
TOTAL	16,555 (54.49%)	13,826 (45.51%)	30,381 (100%)

**Table 2:** NLR (mean, median and IQR) by age and gender.

Age (Years)	Women	Men	*p* Value	Total
18–25	Mean 1.71	Mean 1.69	*p* = 0.118	Mean 1.70
	Median 1.59	Median 1.52		Median 1.56
	IQR: 1.30–1.96	IQR: 1.29–1.93		IQR: 1.30–1.95
26–35	Mean 1.82	Mean 1.71	*p* < 0.0001	Mean 1.77
	Median 1.69	Median 1.56		Median 1.64
	IQR: 1.36–2.12	IQR:1.29–1.99		IQR:1.33–2.07
36–45	Mean 1.89	Mean 1.83	*p* < 0.0001	Mean 1.87
	Median 1.75	Median 1.69		Median 1.73
	IQR: 1.43–2.22	IQR:1.37–2.15		IQR:1.41–2.19
46–55	Mean 1.81	Mean 1.90	*p* < 0.0001	Mean 1.85
	Median 1.68	Median 1.74		Median 1.72
	IQR:1.36–2.12	IQR:1.40–2.24		IQR:1.38–2.17
56–65	Mean 1.77	Mean 2.00	*p* < 0.0001	Mean 1.89
	Median 1.63	Median 1.86		Median 1.74
	IQR:1.34–2.06	IQR:1.49–2.36		IQR:1.41–2.22
>65	Mean 1.89	Mean 2.07	*p* < 0.0001	Mean 1.98
	Median 1.73	Median 1.92		Median 1.82
	IQR:1.39–2.23	IQR:1.51–2.48		IQR:1.44–2.36
TOTAL	Mean:1.83	Mean:1.93	*p* < 0.0001	Mean 1.88
	Median:1.69	Median 1.77		Median1.73
	IQR1.37–2.14	IQR:1.42–2.29		IQR:1.39–2.21

**Table 3:** Published studies of NLR in healthy controls.

	Controls (*N*)	Men/ Women	NLR Mean (Unless Otherwise Specified)	NLR by SEX Mean (Unless Otherwise Specified)	NLR by AGE Mean (Unless Otherwise Specified)
Azab et al. 2014 [37]	9427	M 4625 W 4802	2.15	M 2.19 (95% CI 2.14–2.25) W 2.11 (95% CI 2.07–2.16) *p* > 0.05	non-Hispanic White controls; older age (*p* < 0.0001)
Forget P et al. 2017 [38]	413	NA	1.65 ± 1.47 (SD)	Not Available	Not Available
Meng et al. 2018 [41]	24,029	M 12,660 W 11,369	median 1.72 (IQR 1.37–2.18)	M (median) 1.72 (IQR 1.39, 2.17) W (median) 1.71 (IQR 1.35, 2.18) *p* = 0.010	<65 y (median) 1.71 (IQR 1.36, 2.17) <65 y (median) 1.85 (IQR 1.46, 2.36) *p* < 0.001
Lee JS et al. 2018 [40]	12,160	M 6268 W 5892	1.65 ± 0.79 (SD)	M 1.63 ± 0.76 (SD) W 1.66 ± 0.82 (SD) *p* < 0.05	<19 W 1.654/M 1.627 20–29 W 1.736/M 1.631 30–39 W 1.765/M 1.647 40–49 W 1.754/M 1.620 50–59 W 1.481/M 1.626 60–69 W 1.493/M 1.671 >70 W 1.618/M 1.735 *p* value: NA
Luo H et al. 2019 [42]	5969		(median) 1.77 (2.5th–97.5th % 0.88–4.0)	M (median) 1.75 (2.5th–97.5th % 0.89–3.95) W (median) 1.78 (2.5th–97.5th % 0.87–4.06) *p* = 0.081	<65 y (median) 1.76 (2.5th–97.5th % 0.88, 4.02) >65 y (median) 1.81 (2.5th–97.5th % 0.89, 3.91) *p* = 0.19
Rodriguez Rodriguez E 2020 [44]	1747	M 771 W 976	2.03 ± 0.89 (SD)	M 2.15 ± 0.96 (SD) W 1.93 ± 0.82 (SD) *p* < 0.0001	Not Available
Huguet et al. 2021 [36]	71,873	M 24,334 W 47,539	(median) 1.92 (IQR 1.46–2.58)	Not Available	<19 (median) 1.52 (IQR: 1.19–2.00) 20–29 (median) 1.72 (IQR: 1.29–2.36) 30–39 (median) 2.00 (IQR: 1.50–2.83) 40–49 (median) 1.94 (IQR: 1.52–2.52) 50–59 (median) 1.84 (IQR: 1.43–2.37) 60–69 (median1.91) IQR: 1.47–2.53) 70–79 (median) 2.07 (IQR: 1.56–2.80) 80–89 (median) 2.28 (IQR: 1.67–3.20) >90 (median) 2.44 (IQR: 1.75–3.44) *p* value: NA
Wang J et al. 2021 [39]	404,272	M 205,592 W 198,680		M (median) 1.550 (IQR 1.252–1.933) W (median) 1.587 (IQR 1.272–2.000) *p* < 0.01	18–39 M (median) 1.50/W (median) 1.59 40–49 M (median) 1.55/W (median) 1.68 50–59 M (median) 1.59/W (median) 1.48 60–69 M (median) 1.67/W (median) 1.51 70–79 M (median) 1.74/W (median) 1.60 >80 M (median) 1.78/W (median) 1.76 *p* < 0.0001
Wang Q et al. 2025 [43]	165,504	M 91,936 W 73,568	1.59 ± 0.83 (SD) (median) 1.45 (IQR 1.10–1.88),	M 1.87 ± 0.82 (SD) (median) 1.72 (IQR 1.36–2.19), W 1.91 ± 0.87 (SD) (median) 1.76 (IQR 1.38–2.25) (*p* < 0.0001),	<18 y: 1.59 ± 0.83 (SD) (median) 1.45 (IQR 1.10–1.88) 18–40 y: 1.85 ± 0.81 (SD) (median) 1.71 (IQR 1.35–2.16) 41–60 y: 1.96 ± 0.87 (SD) (median) 1.81 (IQR 1.42–2.31) >61 y 1.99 ± 0.99 (SD) (median) 1.80 (IQR 1.38–2.36) *p* > 0.0001

## Data Availability

Data will be made available upon request to the corresponding author due to ethical limitations.
